# Innovative dual-functional hybrid cationic PEGylated proniosomes as a smart nano-platform for Boosted vaginal delivery: multi-level *in-vitro, ex-vivo,* microbiological, and *in-vivo* studies

**DOI:** 10.3389/fphar.2025.1746918

**Published:** 2026-01-28

**Authors:** Sadek Ahmed, Osama Saher, Heba Attia, Abdurrahman M. Fahmy, Islam M. Adel

**Affiliations:** 1 Department of Pharmaceutics and Industrial Pharmacy, Faculty of Pharmacy, Cairo University, Cairo, Egypt; 2 Department of Laboratory Medicine, Karolinska Institute, Stockholm, Sweden; 3 Department of Cellular Therapy and Allogeneic Stem Cell Transplantation (CAST), Karolinska University Hospital Huddinge and Karolinska Comprehensive Cancer Center, Stockholm, Sweden; 4 Department of Microbiology and Immunology, Faculty of Pharmacy, Cairo University, Cairo, Egypt; 5 School of Life and Medical Sciences, University of Hertfordshire Hosted By Global Academic Foundation, Cairo, Egypt

**Keywords:** biofilm inhibition, ex-vivo permeation, fenticonazole nitrate, hybrid cationic PEGylated proniosomes, Mucoadhesion Enhancement, vaginal drug delivery

## Abstract

**Introduction:** Vaginal candidiasis remains a recurrent fungal infection affecting millions of women worldwide, necessitating innovative local delivery systems to overcome poor drug solubility and mucosal barriers. This study introduces Dual-Functional Hybrid Cationic PEGylated Proniosomes (DHCPP) as a smart nano-platform designed to boost the vaginal delivery of Fenticonazole Nitrate (FTN).

**Methods:** DHCPP systems were fabricated via the coacervation phase separation method and optimized using a full 2^3^ factorial design, achieving a high desirability value of 0.931. The optimized DHCPP exhibited an encapsulation efficiency of 91.61%, particle size of 238.85 nm, and zeta potential of +58.85 mV, ensuring colloidal stability and efficient mucosal interaction.

**Results:** *In-vitro* characterization using TEM and FTIR verified the formation of uniform spherical vesicles and successful FTN encapsulation without chemical incompatibility. Mucoadhesion testing revealed superior adhesive strength, indicating prolonged vaginal residence, while drug release followed a diffusion-controlled pattern. The optimized DHCPP was incorporated into a carbopol-based gel exhibiting pseudo-plastic rheology and pH compatibility with the vaginal environment. Multi-scale *ex vivo* and *in vivo* evaluations revealed a 2.36-fold permeation enhancement compared to FTN gel, consistent with deeper mucosal penetration observed via confocal microscopy. The microbiological assessment indicated a pronounced reduction in MIC and MFC values and a remarkable improvement in biofilm inhibitory effect, highlighting enhanced antifungal efficacy. Histopathological examination verified the mucosal safety of the optimized gel.

**Conclusion:** The developed DHCPP represents an innovative, multifunctional, and biocompatible delivery system offering enhanced vaginal permeation, prolonged retention, and potent antifungal activity for the effective management of vaginal candidiasis.

## Highlights

- Innovative Dual-Functional Hybrid Cationic PEGylated Proniosomes (DHCPP) were developed to enhance vaginal delivery of Fenticonazole Nitrate (FTN).
- Formulation optimization using a full 2^3^ factorial design achieved a high desirability value (0.931), producing stable nano-sized vesicles (238.85 nm) with high EE% (91.61%) and strong positive ZP (+58.85 mV).
- FTIR and TEM analyses confirmed molecular compatibility, successful PEGylation, and the formation of uniform spherical vesicles ensuring effective drug entrapment.
- DHCPP revealed enhanced mucoadhesion, diffusion-controlled release, prolonged vaginal retention, supported by pseudo-plastic rheology and physiological pH compatibility.
- Comprehensive *in-vitro, ex-vivo*, microbiological, and *in-vivo* evaluations demonstrated a 2.36-fold permeation enhancement, pronounced biofilm inhibition, and significant reduction in MIC and MFC values.
- Histopathological findings verified mucosal safety, positioning DHCPP as an innovative, biocompatible, and clinically promising nanocarrier for effective management of vaginal candidiasis.

## 1 Introduction

Fungal infections remain a persistent and expanding global health challenge, particularly among immunocompromised individuals and women of reproductive age. Among the diverse pathogenic fungi, *Candida* species are the predominant culprits, responsible for approximately 90% of fungal infections worldwide (Campelo et al., 2021). Vaginal candidiasis, primarily induced by *Candida albicans*, represents one of the most prevalent gynecological disorders, affecting around three out of every four women will experience an episode during their lifetime, and nearly half of them may suffer from repeated recurrences over time (Bhosale et al., 2025). Such alarming recurrence rates underscore the inadequacy of conventional antifungal therapies in providing durable clinical resolution. The etiology of vaginal candidiasis is multifactorial, involving hormonal fluctuations linked to oral contraceptive use, uncontrolled diabetes, and, most prominently, the widespread administration of broad-spectrum antibiotics that disrupt the protective vaginal microbiota (Chassot et al., 2008). The ensuing dysbiosis compromises mucosal immunity, enabling fungal overgrowth and recurrent infections. Clinically, patients present with pruritus, burning, irritation, and profuse malodorous discharge, all of which collectively diminish quality of life and psychological wellbeing (Ahmed et al., 2025a). Currently, azole antifungals, particularly fluconazole, remain the cornerstone of therapy due to their inhibition of the fungal cytochrome P450-dependent ergosterol synthesis pathway (Ahmed et al., 2025b). However, increasing reports of azole resistance, coupled with systemic adverse effects such as gastrointestinal distress, hepatotoxicity, and cardiac arrhythmias, have intensified the demand to achieve enhanced site-specific efficacy with improved safety and minimal systemic exposure (Picheta et al., 2024). In this context, the vaginal route offers an attractive and targeted alternative for antifungal delivery. It allows site-specific administration, enhanced drug concentration at the infection locus, and reduced systemic exposure. Nevertheless, challenges such as poor drug solubility, rapid mucosal clearance, and limited penetration across the vaginal epithelium necessitate the development of advanced nanocarrier systems (Albash et al., 2020).

The accelerating advancements in nanotechnology have revolutionized the pharmaceutical landscape, providing transformative approaches designed to address and mitigate the drawbacks associated with conventional drug delivery systems (Farag et al., 2025). Emerging nanocarrier-based systems enable precise modulation of drug pharmacokinetics and overall bio-distribution, leading to improved treatment effectiveness with reduced risk of systemic side effects (Elmahboub et al., 2025). These next-generation delivery platforms are meticulously engineered to achieve controlled and site-specific drug release, ensuring optimal spatiotemporal availability of active pharmaceutical ingredients and improved patient compliance (Ahmadi et al., 2023). The present study introduces Dual-Functional Hybrid Cationic PEGylated Proniosomes (DHCPP) as an innovative nano-platform engineered to enhance the vaginal delivery of Fenticonazole Nitrate (FTN). This hybrid system strategically combines cationic charge-induced mucoadhesion with PEGylation-driven stability and biocompatibility, yielding a synergistic architecture that improves mucosal residence, permeability, and antifungal efficacy. The system is primarily composed of di-dodecyl-dimethyl-ammonium bromide (DDAB), a cationic quaternary ammonium surfactant that constitutes the structural backbone of the vesicular membrane (Albash et al., 2021). The positive surface charge imparted by DDAB facilitates ionic attraction with the anionic mucosal surface, resulting in stronger adherence and extended residence time within the vaginal environment. Phosphatidylcholine (PC), a biocompatible phospholipid, was incorporated as a bilayer-forming agent to mimic natural biological membranes, facilitating drug encapsulation and controlled release while maintaining structural integrity (Ahmed et al., 2024a). To further reinforce membrane rigidity and minimize premature drug leakage, cholesterol was included to intercalate within the lipid bilayer, conferring both mechanical strength and thermodynamic stability to the vesicles (Younes et al., 2024). Additionally, Pluronic® L121, a non-ionic triblock copolymer, acts as a surface-active stabilizer that enhances flexibility and prevents vesicle aggregation, contributing to improved dispersibility and sustained release behavior (Ahmed et al., 2025c). Complementing this design, PEGylation, the covalent attachment of polyethylene glycol (PEG) chains, was introduced to the vesicular surface to impart steric stabilization and improve the nano-system’s resistance to enzymatic degradation. PEGylation also mitigates immunogenic responses and enhances residence time within the mucosal milieu, ensuring prolonged therapeutic exposure and consistent antifungal action (Fahmy et al., 2025; Megahed et al., 2022).

Fenticonazole Nitrate (FTN) is a synthetically derived imidazole-based antifungal agent with broad-spectrum activity known for its concentration-dependent activity (Ahmed et al., 2023). At sub-inhibitory concentrations, FTN demonstrates a potent biofilm-suppressive effect, disrupting early fungal adhesion and thereby minimizing the likelihood of infection recurrence and chronic colonization (Sanguinetti et al., 2019). Conversely, at higher concentrations, FTN exerts a fungicidal mechanism by enhancing membrane permeability and causing disruption of the fungal cell membrane architecture, which ultimately results in irreversible cell rupture and death (Ahmed et al., 2022). FTN exhibits good thermal stability in its solid crystalline state, with a reported melting point in the range of approximately 135 °C–137 °C, indicating a stable crystal lattice capable of withstanding moderate thermal conditions. This relatively high melting point suggests that FTN can tolerate the temperatures commonly employed during formulation and processing without undergoing thermal degradation (Ahmed et al., 2025a; Nemr et al., 2025). Clinical investigations have confirmed the therapeutic effectiveness of FTN through short-course vaginal ovule regimens, which notably improved patient adherence and tolerability compared to conventional azole therapies (Lawrence et al., 1990). However, despite its advantageous pharmacodynamic properties, FTN’s poor aqueous solubility (<0.1 mg/mL) and limited systemic bioavailability significantly restrict its therapeutic potential and consistent local retention (Nemr et al., 2024). These challenges emphasize the necessity for a next-generation localized delivery system that can improve drug solubility, facilitate deeper mucosal diffusion, and maintain prolonged antifungal effectiveness.

The objective of this research was to design and optimize FTN-loaded DHCPP formulations with the goal of attaining maximal encapsulation efficiency and surface charge stability, alongside reduced particle size and a narrow poly-dispersity index, ensuring a homogeneous nanoscale distribution. A full 2^3^ factorial experimental design was employed through Design Expert® software (v13, Stat-Ease Inc., Minneapolis, USA) to systematically explore formulation variables and determine the most optimized composition. Before incorporating the optimized formula into a gel, comprehensive *in-vitro* evaluations were performed. These included drug release studies, surface morphology using TEM, and interaction analysis by FTIR. In addition, mucoadhesion testing was carried out to confirm the enhanced adhesive properties of the cationic PEGylated system, and stability testing was performed under refrigerated conditions for 3 months to ensure formulation integrity. The optimized DHCPP were then incorporated into a carbopol-based gel designed for vaginal application. The prepared gel was examined for rheological behavior and pH compatibility to confirm suitability for mucosal use. Ultimately, *ex-vivo* permeation experiments combined with *in-vivo* confocal laser scanning microscopy (CLSM) imaging demonstrated enhanced vaginal tissue penetration, and histological examination further validated the biocompatibility and safety of the developed formulation on the mucosal surface.

## 2 Materials and methodology

### 2.1 Materials

FTN, possessing a molecular weight of 518.41 g/mol and a purity exceeding 99%, was kindly supplied by Andalous Pharmaceutical Company. PC; (egg yolk, ∼60%), DDAB (98% purity, melting point 157-162 ℃ ), PEG 400 (M_wt_ = 380-420), Pluronic L121 (viscous liquid, viscosity 1200 cP, average M_n_ = 4,400), Cholesterol (Ch; ≥ 99% purity, white powder, melting point 147-149 ℃ ) were obtained from Sigma-Aldrich (St. Louis, MO, USA). A semipermeable cellulose dialysis membrane with a molecular weight cutoff of 14,000 Da was procured from Sigma-Aldrich (St. Louis, MO, USA). All other chemicals and solvents utilized in the study were of analytical grade and used without further purification.

### 2.2 Design of experiments

To methodically evaluate how different formulation parameters affect the physicochemical behavior of the prepared FTN–loaded DHCPPs, a full 2^3^ factorial experimental design was employed utilizing Design-Expert® software (version 13; Stat-Ease Inc., Minneapolis, MN, USA).This statistical tool was employed for its efficiency in simultaneously evaluating multiple factors and their possible interactions while minimizing experimental runs (Ahmed et al., 2025d). Based on preliminary trials, three key formulation parameters were chosen as independent variables: the ratio of DDAB to drug (Factor A), the PEG-to-DDAB ratio (Factor B), and the PC-to-PEG ratio (Factor C). Each parameter was examined at two distinct levels to provide a comprehensive understanding of its impact on formulation performance. The evaluated dependent variables comprised encapsulation efficiency (EE%; Y_1_), particle size (PS; Y_2_), poly-dispersity index (PDI; Y_3_), and zeta potential (ZP; Y_4_), which collectively serve as key determinants (pre-optimization measurements) of the formulation’s drug encapsulation capability, nanoscale uniformity, and overall colloidal stability. This factorial approach enabled the identification of significant main effects, facilitating the optimization of formulation composition toward achieving high EE%, nanoscale particle size, and stable charge characteristics (El Hassab et al., 2025; Ahmed et al., 2025e). A detailed overview of the experimental layout, outlining the factor levels alongside the corresponding measured responses, is provided in Table 1, whereas Table 2 summarizes the obtained results for all formulated batches.

**TABLE 1:** 2^3^ full factorial experimental design highlighting different levels of independent variables and targeted optimization criteria for the responses.

Factor (independent variable)	Level	
	−1	+1
A: DDAB: Drug ratio B: PEG: DDAB ratio C: PC: PEG ratio	2.5 1.5 1	5 3 2
Response (dependent variable)	Desirability constraints	
Y1: EE % Y2: PS (nm) Y3: PDI Y4: ZP (absolute value) (mV)	Maximize Minimize Minimize Maximize	

Abbreviations: DDAB, Di-dodecyl-di-methyl-ammonium bromide; PEG, Polyethylene glycol 400; PC, phosphatidylcholine; EE%, entrapment efficiency percentage; PS, particle size; PDI, Poly-dispersity index; ZP, zeta potential.

**TABLE 2:** Composition and Characterization of the prepared FTN-loaded Hybrid Cationic PEGylated Proniosomes (mean ± SD).

Formula	Factors			Responses				
	A: DDAB: drug ratio	B: PEG: DDAB ratio	C: PC: PEG ratio	Y1: EE % (mean ± SD)	DL% (mean + SD)	Y2: PS (nm) (mean ± SD)	Y3: PDI (mean ± SD)	Y4: ZP (mV) (mean ± SD)
F1	2.5	1.5	2	83.63 ± 1.63	3.79 ± 0.05	256.05 ± 2.33	0.25 ± 0.01	55.50 ± 1.27
F2	5	3	1	78.42 ± 2.62	2.05 ± 0.06	230.90 ± 0.14	0.22 ± 0.02	50.05 ± 0.78
F3	2.5	3	1	72.34 ± 2.37	3.37 ± 0.02	253.75 ± 4.31	0.23 ± 0.01	45.50 ± 0.57
F4	2.5	1.5	1	74.51 ± 3.12	4.61 ± 0.04	283.60 ± 1.13	0.27 ± 0.03	50.05 ± 0.78
F5	5	3	2	89.40 ± 2.32	1.60 ± 0.02	226.65 ± 3.04	0.24 ± 0.02	58.20 ± 3.11
F6	5	1.5	1	80.91 ± 4.15	3.01 ± 0.09	240.20 ± 5.23	0.20 ± 0.01	52.00 ± 1.13
F7	5	1.5	2	91.61 ± 1.98	2.49 ± 0.08	238.85 ± 3.46	0.22 ± 0.01	58.85 ± 2.47
F8	2.5	3	2	87.32 ± 1.56	2.64 ± 0.08	241.80 ± 1.56	0.22 ± 0.01	55.60 ± 1.70

FTN, ch, and Pluronic L121 were added to all formulations at constant amounts (10, 50, and 50 mg, respectively).

Abbreviations: DL%, drug loading percentage; FTN, fenticonazole; PEGylated, Polyethylene glycol 400-coated; DDAB, Di-dodecyl-di-methyl-ammonium bromide; PC, phosphatidylcholine; EE %, entrapment efficiency percentage; PS, particle size; PDI, poly dispersity index; ZP, zeta potential; Ch, Cholesterol.

### 2.3 Development of FTN-loaded DHCPP

The FTN-loaded DHCPPs were fabricated using a modified coacervation phase separation method, as adapted from previously reported with slight modifications (Ahmed et al., 2025f; Khatoon et al., 2017). In brief, accurately weighed quantities of FTN (10 mg), cholesterol (50 mg), and Pluronic L121 (50 mg) were incorporated in all formulations as fixed components, whereas the proportions of DDAB, PEG, and PC were varied according to the factorial design parameters. All components were solubilized in a tightly closed glass vial containing 2 mL of a methanol and acetone mixture prepared in a 1:1 volume ratio. The mixture was maintained at 60 °C in a water bath and sonicated (Model SH 150–41, MTI Corporation, Richmond, CA) for 30 min to obtain a homogeneous clear dispersion. Afterward, three drops of phosphate-buffered saline (PBS, pH 7.4), pre-warmed to the required temperature, were gradually introduced while maintaining continuous sonication for a further 15 min. The resulting solution was left to slowly reach ambient temperature and then stored overnight. Before further characterization, the stored formulations were preheated to 60 °C for 1 minute, then hydrated with 10 mL of double-distilled water under vortex agitation for 5 min to yield uniform, nano-sized niosomal dispersions (Younes et al., 2024).

### 2.4 Analysis of the developed FTN-loaded DHCPP

#### 2.4.1 Evaluation of encapsulation efficiency (EE%) and drug loading (DL%)

The encapsulation efficiency (EE%) of the FTN-loaded DHCPP formulations was assessed indirectly by ultracentrifugation, measuring the amount of drug remaining in the aqueous phase that was not encapsulated (Nemr et al., 2025). Accurately measured aliquots of the freshly prepared formulations (1 mL, equivalent to 1000 µg FTN) were transferred into centrifuge tubes. The samples were then centrifuged at 21,000 rpm for 1 h at 4 °C using a refrigerated centrifuge (Model 8,880, Centurion Scientific Ltd., West Sussex, UK). This step ensured complete sedimentation of the vesicular fraction, leaving the supernatant containing the free, non-encapsulated FTN. The transparent supernatant was gently collected, suitably diluted to a final volume of 10 mL using methanol, corresponding to a 1:10 (v/v) dilution ratio, and its absorbance was measured at 252 nm using a UV–visible spectrophotometer (UV-1601 PC, Shimadzu, Kyoto, Japan). Quantification was performed against a validated calibration curve (*R* ^2^ = 0.9997, n = 3) to ensure analytical accuracy and reproducibility. For drug loading (DL%) measurement, a methanolic solution of the sample volume (0.5 mL; equivalent to 500 µg FTN) was prepared and measured for FTN content. The encapsulation efficiency (EE%) and drug loading (DL%) was subsequently determined using the Equations 1, 2 (Ahmed et al., 2025a; Teama et al., 2025; Herdiana et al., 2024):

EE%=total amount of the drug−free drugdrug″ in the supernatant″total amount of the drug×100 (1)

$$\text{DL}\%=\frac{actual\;drug\;amount}{actual\;drug\;amount+weight\;of\;used\;components}\times 100$$ (2)

#### 2.4.2 Evaluation of particle size, poly-dispersity index and surface charge

The particle size (PS), poly-dispersity index (PDI), and zeta potential (ZP) of the developed FTN-loaded DHCPP formulations were analyzed to evaluate their colloidal characteristics and stability. Dynamic Light Scattering (DLS) analysis was performed using a Zetasizer Nano ZS instrument (Malvern Instruments Ltd., Worcestershire, UK) at 25 °C ± 1 °C with a backscattering angle of 173° (Ahmed et al., 2025g). Before measurement, 50 μL of the freshly prepared sample was diluted with double-distilled water to a final volume of 10 mL and mixed gently using a vortex mixer to ensure a uniform, translucent dispersion suitable for accurate light scattering. The particle size provided insight into the nanoscale dimensions of the vesicles, The polydispersity index (PDI) indicated the degree of uniformity in particle size distribution, with smaller PDI values corresponding to a more uniform vesicular population (Albash et al., 2025a). The zeta potential, determined through electrophoretic mobility within the same instrument, indicated the surface charge of the vesicles and was used to predict colloidal stability and the potential for electrostatic interaction with the vaginal mucosa (El-Naggar et al., 2026). All measurements were carried out in triplicate, and the data are presented as mean ± standard deviation to guarantee reliability and reproducibility of the analysis (Bazaz et al., 2021).

### 2.5 Choice of best-performing DHCPP

FTN-loaded DHCPP formulations were systematically optimized using Design-Expert® software (version 13, Stat-Ease Inc., Minneapolis, USA) to determine the formulation with the most favorable physicochemical characteristics. The optimization parameters were established to achieve maximum encapsulation efficiency (EE%; Y_1_) and high absolute zeta potential (ZP; Y_4_), while minimizing particle size (PS; Y_2_) and keeping the polydispersity index (PDI; Y_3_) within a suitable range to ensure uniform nanoscale distribution and stable colloidal behavior (Ahmed et al., 2024b). Analysis of variance (ANOVA) was employed to statistically examine the significance of the model terms and the interactions between formulation variables. A numerical multi-response optimization approach was then applied, where each response was transformed into an individual desirability function ranging from 0 (least desirable) to 1 (most desirable) (Ahmed et al., 2025e). These individual functions were subsequently integrated into a composite desirability index, allowing simultaneous consideration of all targeted parameters. The formulation displaying the highest overall desirability value was identified as the optimized system and selected for confirmatory evaluation (Albash et al., 2025b). The close agreement between predicted and experimental values, expressed as a low percentage deviation, verified the accuracy, stability, and predictive capability of the optimization model was determined using Equation 3 (Albash et al., 2025c).

$$\%\text{ Deviation}=\left(\left|\text{Predicted value}-\text{Observed value}\right|/\left(\text{Observed value}\right)\right)\times 100$$ (3)

### 2.6 Evaluation of the selected DHCPP

#### 2.6.1 TEM visualization

TEM (JEOL JEM-2100, Frankfurt, Germany) was used to scan the surface morphology and structure of the optimized DHCPP formulation, confirming vesicle formation and uniform nanoscale characteristics (Elgendy et al., 2024). Before imaging, the chosen formulation was diluted with deionized water to ensure adequate dispersion and reduce vesicle aggregation. First, the sample was properly diluted, and a drop was taken and gently applied onto a carbon-coated copper grid. The grid was left to dry in an open atmosphere at room temperature. To improve contrast and clearly visualize the vesicle boundaries, the sample was negatively stained with a 0.1% (w/v) phosphotungstic acid solution and allowed to dry for additional 15 min. The prepared specimens were subsequently examined at an accelerating voltage of 80 kV, enabling detailed visualization of the vesicle morphology, surface smoothness, and structural integrity (Tawfik et al., 2025).

#### 2.6.2 FT-IR analysis

FTIR analysis was carried out to investigate potential physicochemical interactions among the components of the optimized FTN-loaded DHCPP and to confirm successful drug encapsulation within the nanocarrier matrix. The newly prepared optimum formulation was first frozen at −20 °C and subsequently lyophilized at −45 °C using a Novalyphe-NL 500 freeze-dryer (Savant Instruments, NY, USA) to obtain a completely dry powder (Ahmed et al., 2022). The effect of lyophilization on the nanoscale characteristics of the optimized formulation was evaluated by measuring particle size and poly-dispersity index (PDI) before and after freeze-drying. After that, an accurately weighed portion of the lyophilized sample (2 mg) was thoroughly mixed with anhydrous potassium bromide (KBr) and pressed into a clear pellet using hydraulic pressure (Ahmed et al., 2025h). The FTIR spectra were acquired at room temperature using a Shimadzu FTIR spectrophotometer (Model 8400 S, Kyoto, Japan) across a wavenumber range of 4,000–400 cm^-1^ (Elgendy et al., 2023). For comparative analysis, spectra of pure FTN, DDAB, PC and cholesterol were also obtained under identical conditions.

#### 2.6.3 *In-vitro* release study

*In vitro* drug release was evaluated using the dialysis bag diffusion method to compare the release behavior of the optimized FTN-loaded DHCPP formulation with that of the plain drug suspension. For the preparation of the FTN suspension, the drug was uniformly dispersed in distilled water containing methylcellulose as a suspending agent. Methylcellulose was chosen owing to its biological inertness, excellent suspending properties, and minimal interference with drug stability or analytical assessment. The suspension was prepared under continuous stirring to ensure complete homogeneity and was freshly prepared prior to each use to maintain consistency and reliability.

Dialysis membranes with a molecular weight cut-off of 12–14 kDa were pretreated by soaking overnight in phosphate-buffered saline (PBS, pH 4.5) and subsequently filled with a precisely measured amount of the optimized formulation (1 mL, equivalent to 1000 µg FTN) or an equivalent drug suspension. Each dialysis membrane was tightly sealed and placed into 50 mL of release medium, composed of phosphate-buffered saline (PBS, pH 4.5) and absolute ethanol in a 3:1 (v/v) ratio, to ensure sink conditions (Ahmed et al., 2025b). The assemblies were placed in a thermostatically controlled shaking water bath (Unimax, IKA, Germany) maintained at 37 °C ± 0.2 °C and agitated at 100 rpm to simulate physiological conditions (Weng et al., 2020). At specific time intervals (0.5, 1, 2, 4, 6, and 8 h), 3 mL samples were collected and promptly replaced with an equal volume of fresh pre-warmed medium. The collected samples were examined using spectrophotometry to quantify the cumulative release of FTN. The results were plotted as cumulative percentage of drug released versus time, and Q_2_h and Q_8_h values were calculated and compared with those from the plain drug suspension. The release profiles were analyzed using zero-order, first-order, Korsmeyer–Peppas, and Higuchi kinetic models, with the correlation coefficient (*R* ^2^) employed to determine the model that most accurately represented FTN release from the optimized DHCPP formulation (Ahmed et al., 2025c; Abdelhakeem et al., 2025).

#### 2.6.4 Mucoadhesion assessment

The mucoadhesive properties of the optimized FTN-loaded DHCPP formulation were investigated by examining its electrostatic interactions with mucin. A 1% (w/v) mucin suspension was combined with an equal volume of the optimized DHCPP formulation in a 1:1 (v/v) ratio (Nemr et al., 2024). The mixture was carefully agitated using a magnetic stirrer for 5 min to ensure homogeneity, followed by overnight equilibration at room temperature to allow sufficient interaction between mucin glycoproteins and the positively charged vesicular surface (Tawfik et al., 2025). The zeta potential (ZP) of the mucin dispersion and the optimized DHCPP formulation was determined individually and compared to that of the mucin–DHCPP mixture employing a Zetasizer (Model ZEN3600, Malvern Instruments Ltd., Worcestershire, UK). A significant change in the ZP of the mixture compared to the individual components was interpreted as an indication of strong electrostatic interaction and effective mucin binding, confirming the enhanced mucoadhesive behavior of the optimized formulation which is an essential attribute for prolonged retention and improved drug residence at the vaginal mucosa (Albash et al., 2021; Ahmed et al., 2025d).

#### 2.6.5 Short term stability assessment

The influence of storage on the physicochemical stability, as well as the release behavior, of the selected FTN-loaded DHCPP formulation was studied (Fahmy et al., 2025). Newly prepared samples were evaluated for encapsulation efficiency (EE%, Y_1_), particle size (PS, Y_2_), zeta potential (ZP, Y_4_), and *in-vitro* drug release profiles. After characterization, the samples were placed in tightly sealed amber glass vials and stored at 4 °C–8 °C for 3 months to protect them from light- and temperature-induced degradation (Sayed et al., 2021). This duration is widely used in preliminary assessments of nanoscale systems and has been shown in previous studies to reliably predict the possible stability trends of similar formulations (Younes et al., 2024; Ahmed et al., 2025f). After the 3-month storage period, the same parameters were re-evaluated under identical experimental conditions to detect any potential alterations in vesicular integrity or drug retention. One-way ANOVA was applied to statistically compare EE%, PS, and ZP between freshly prepared and stored samples, assessing the significance of any observed differences (Al-Mahallawi et al., 2017). Additionally, the *in-vitro* release profiles of both formulations were compared by calculating the similarity factor (ƒ_2_), using Equation 4 (Emad Eldeeb et al., 2019):

fn=50.⁡log⁡{1+1n∑t=1nRt−Tt2−0.5.100 (4)

where n is the number of sampling points, R_t_ represents the cumulative percentage of FTN released from the fresh sample at time t, and T_t_ denotes the corresponding release from the stored sample. An ƒ_2_ value between 50 and 100 indicated no significant difference in release behavior, confirming that the optimized formulation retained its colloidal stability, encapsulation capacity, and release performance throughout refrigerated storage (Abdelbary et al., 2016; Ahmed et al., 2025).

### 2.7 Microbiological assay

#### 2.7.1 Determination of minimum inhibitory concentration (MIC)

The minimum inhibitory concentration (MIC) of the optimized DHCPP formulation and the pure drug suspension was determined using the broth microdilution assay, performed in accordance with the Clinical and Laboratory Standards Institute (CLSI) guidelines (Humphries et al., 2018). A series of two-fold serial dilutions of each tested sample was prepared within a concentration range of 1000–0.976 μg/mL, using double-strength Sabouraud Dextrose Broth (SDB) as the nutrient medium. Precisely 150 µL of each dilution was transferred into sterile 96-well microtiter plates with U-shaped wells, followed by inoculation with 30 µL of a standardized *C. albicans* (ATCC 60193) suspension containing approximately 10^5^–10^6^ CFU/mL. Appropriate controls were included: a positive control containing inoculum without treatment to confirm fungal viability, and a negative control containing un-inoculated broth to ensure sterility of the medium. Immediately, the plates were placed in an incubator at 28 °C ± 2 °C for 48 h. Following incubation, fungal growth inhibition was evaluated both visually and spectrophotometrically by measuring optical density at 600 nm using a microplate reader (Biotek Synergy 2, SLFA model, USA). The MIC value was defined as the lowest concentration of the tested formulation exhibiting no visible growth or turbidity compared to the control wells. All experiments were carried out in biological and technical triplicates to ensure data reproducibility.

#### 2.7.2 Determination of minimum fungicidal concentration (MFC)

Serial two-fold dilutions of both the pure drug suspension and the optimized DHCPP were prepared and inoculated with 30 μL of a *C. albicans* culture standardized to an inoculum density of 10^5^–10^6^ CFU/mL. The mixtures were placed in 96-well microplates and incubated at 28 °C ± 2 °C for 48 h. Following incubation, 10 μL samples from each well were aseptically transferred and spotted onto Sabouraud Dextrose Agar (SDA) plates. These plates were further incubated under the same conditions (28 °C ± 2 °C for an additional 48 h). The minimum fungicidal concentration (MFC) was identified as the lowest concentration that exhibited complete inhibition of visible fungal growth. All assays were conducted in triplicate, covering both biological and technical replicates to ensure reproducibility (Ahmed et al., 2025b).

#### 2.7.3 Determination of biofilm inhibitory effect

The anti-biofilm potential of both the plain drug suspension and the optimized formulation was assessed against *C. albicans* standard strain using the microtiter plate method as previously described (O'Toole, 2011; Van Dijck et al., 2018). Briefly, 75 μL of the optimized DHCPP was combined with an equal volume (75 μL) of *C. albicans* inoculum (approximately 10^8^ CFU/mL in Sabouraud Dextrose Broth, SDB) in sterile, non-pyrogenic, flat-bottom 96-well polystyrene plates. The resulting final concentrations within the wells corresponded to ^1^/_16_, ^1^/_8_, ¼, and ½ X, where X denotes the previously determined minimum inhibitory concentration. The plates were incubated statically at 28 °C ± 2 °C for 48 h, allowing biofilm development. Following incubation, the optical density at 600 nm (OD_600_) was measured via a spectrophotometric plate reader (Synergy 2, USA) to quantify planktonic growth. The same procedure was repeated for drug suspension and the results were compared to that of the optimized formulation.

After measuring planktonic growth, the wells were gently decanted, rinsed twice with sterile phosphate-buffered saline (PBS) to remove the supernatant, and air-dried thoroughly. The remaining adherent biofilms were stained with 175 μL of 0.5% crystal violet for 30 min at room temperature. Excess dye was carefully removed by 3 washing cycles of the plates with sterile distilled water, followed by complete drying. The bound dye, representing the residual biofilm biomass, was then solubilized with 200 μL of 95% ethanol and incubated for 15 min under gentle agitation (110 rpm). The absorbance of the resulting solution was determined at 570 nm (OD_570_) and normalized to the corresponding OD_600_ values of planktonic cultures. All experiments were conducted in triplicate, including both biological and technical replicates, with positive and negative controls incorporated to validate the assay. The statistics were performed in version 4.1.2 of R and visualized in Rstudio (Racine, 2012). The percentage of biofilm inhibition was subsequently calculated according to the following Equation 5 (Ahmed et al., 2025e):

$$\text{Biofilm inhibition }\%=\frac{\text{OD}\;\text{Control}-\text{OD}\;\text{Test}}{\text{OD}\;\text{Control}}\times 100$$ (5)

### 2.8 Development and characterization of optimum DHCPP gel

#### 2.8.1 Development of DHCPP gel

Following the selection of the optimized DHCPP formulation, a carbopol-based gel was developed to facilitate vaginal application and ensure prolonged drug retention at the target site. To prepare the gel, an accurately weighed amount of Carbopol 940 (1% w/w) was gradually dispersed into the freshly prepared optimized DHCPP under continuous magnetic stirring (300 rpm) at 40 °C, ensuring uniform polymer hydration and avoiding lump formation (Shamma et al., 2019). Once a clear dispersion was obtained, triethanolamine (TEA) was cautiously introduced dropwise to neutralize the mixture and trigger gelation through pH adjustment. The prepared gel was subsequently allowed to stand overnight at room temperature to ensure complete polymer swelling and stabilization of the network structure. The resulting transparent, homogenous DHCPP gel exhibited smooth texture and was stored under refrigerated conditions (4 °C–8 °C) until further evaluation of its physicochemical and performance attributes (Ahmed et al., 2025b; Younes and Habib, 2022).

#### 2.8.2 Rheological study

The rheological characteristics of the optimized DHCPP gel were systematically evaluated to determine its flow profile, and suitability for vaginal application. Measurements were carried out using a rotational cone-and-plate rheometer (Brookfield DV3THB, spindle CPE-41) maintained at a controlled temperature of 25 °C ± 2 °C. An accurately weighed 0.5 g sample of the gel was placed on the plate, and the shear rate was gradually increased from 0.5 to 100 rpm, with a 10-s equilibration period at each speed increment to ensure steady-state readings (Ahmed et al., 2025e). Only torque values within the 10%–100% range were included in the analysis to maintain precision and minimize measurement error. The resulting data were plotted to construct a comprehensive flow curve correlating shear stress, shear rate, and apparent viscosity. Rheological data were fitted using the Ostwald–de Waele (Power Law) model due to its proven accuracy in describing the shear-dependent behavior of non-Newtonian vesicular and semisolid systems. The model is widely reported in the literature for proniosomal and vaginal formulations and showed an excellent fit to the experimental data in the present study (Ahmed et al., 2025a; Ahmed et al., 2025b). The experimental data were fitted to the following Equation 6 (Ahmed et al., 2025):

$$\tau =K\gamma^{n}$$ (6)

where Ʈ represents shear stress, γ denotes shear rate, K is the consistency index, and n is the flow behavior index. The value of n was used to classify the type of flow: n = 1 corresponds to Newtonian behavior, n < 1 indicates pseudoplastic (shear-thinning) flow, and n > 1 reflects dilatant (shear-thickening) properties (Fahmy et al., 2018). To gain deeper insight into the viscoelastic and non-Newtonian characteristics of the system, additional rheological models (Bingham, Casson, and Carreau) were also applied. The coefficient of determination (*R* ^2^) was used to evaluate model fitting accuracy and identify the best representation of the gel’s flow dynamics (Younes et al., 2024).

#### 2.8.3 pH measurement

The pH of the optimized formulation was assessed to verify its physiological compatibility and ensure safe application at the intended administration site without inducing irritation or discomfort. Measurements were conducted using a calibrated benchtop pH meter (Jenway®, Model 3510, Cole-Parmer®, Illinois, USA). A defined quantity of the gel was dispersed in distilled water (1:10 w/v) to facilitate consistent readings, and measurements were performed in triplicate at room temperature. The results were recorded as mean ± standard deviation (SD) (Ahmed et al., 2024b; Fouda et al., 2018).

### 2.9 Animal studies

#### 2.9.1 Ethical approval

Animal studies were conducted following strict ethical and scientific standards to ensure animal welfare and data reliability. The study protocol received prior approval from the Research Ethics Committee of the Faculty of Pharmacy, Cairo University, Egypt (Approval No. PI 3771). All procedures adhered to the ethical principles outlined in the Guide for the Care and Use of Laboratory Animals (U.S. National Institutes of Health, NIH Publication No. 85–23, revised 2011) and complied with the ARRIVE guidelines for transparent reporting of *in vivo* experiments.

Adult female albino rabbits (weighing 2 ± 0.5 kg) were selected as the biological model (Ahmed et al., 2025a). Animals were housed individually in well-aerated cages under strict laboratory conditions including a temperature of 25 °C ± 2 °C, a 12 h-light/dark cycle, and properly regulated humidity. Throughout the experimental period, rabbits were provided *ad libitum* access to a standard laboratory diet and clean drinking water. Prior to study initiation, each rabbit underwent a veterinary health screening to confirm the absence of infection or inflammatory disorders that could interfere with the results.

#### 2.9.2 *Ex-vivo* permeation study

Six Adult female albino rabbits (2 ± 0.5 kg) were randomly divided into two groups (n = 3). They were anesthetized using I.M. injection of ketamine (35 mg/kg) and xylazine (5 mg/kg) to facilitate tissue excision (Sayed et al., 2020). Following anesthesia, the vaginal canal was carefully dissected, isolated from adjacent tissues, and thoroughly defatted with isopropyl alcohol. The membrane was then gently washed several times with saline and deionized water to eliminate residual contaminants, after which it was stored at −20 °C until further use in permeation experiments (Ahmed et al., 2025a; Satapathy et al., 2023). The *ex vivo* permeation behavior of the optimized DHCPP gel was examined using a modified Franz diffusion cell specifically designed to mimic physiological vaginal conditions. The apparatus consisted of two compartments, a donor and a receptor cell, separated by the excised rabbit vaginal membrane, oriented with the epithelial surface towards the donor compartment to simulate *in vivo* orientation. An accurately weighed amount of the optimized DHCPP gel (equivalent to 1000 µg FTN) was applied to the donor chamber. The receptor compartment contained 25 mL of phosphate-buffered saline (PBS, pH 4.5) mixed with absolute ethanol (3:1 v/v) to maintain sink conditions (Ahmed et al., 2025b). The system was maintained at a controlled temperature of 37 °C ± 0.2 °C and continuously agitated at 100 rpm. At predetermined intervals (1, 2, 4, 6, 8, and 10 h), 1 mL samples were withdrawn from the receptor medium and replenished with equal volumes of fresh pre-warmed medium to maintain equilibrium. Collected samples were first filtered through a 0.22 µm nylon filter to remove any biological particulates and then analyzed spectrophotometrically (Ahmed et al., 2025c; Ahmed et al., 2025f). UV spectrophotometry has been widely reported in the literature as a validated and reliable method for determination, and several studies have successfully employed this technique for similar nanoscale formulations (Ahmed et al., 2024a; Mohy et al., 2026).

To provide a comparative analysis, a similar quantity of plain FTN gel (negative control) was similarly tested under identical experimental conditions. The cumulative drug permeation per unit area (Q, μg/cm^2^) was plotted against time to derive the permeation profile, from which key kinetic parameters, namely, the maximum flux (Jmax) and enhancement ratio (ER) were calculated using Equations 7, 8 (Ahmed et al., 2024a; ElMeshad and Mohsen, 2016):

$$J_{\max}=\frac{Amount\;of\;drug\;permeated}{Time\;X\;Area}$$ (7)

$$\text{ER}=\frac{J_{\max\;}\text{of}\;\text{optimum}\;\text{formula}}{J_{\max}\;\text{of}\;\text{control}}$$ (8)

#### 2.9.3 Confocal laser scanning microscopy (CLSM)

The *in vivo* vaginal permeation profile of the optimized DHCPP gel was evaluated using Confocal Laser Scanning Microscopy (CLSM) to visualize and quantify the extent of mucosal penetration. To enable fluorescence-based tracking, Rhodamine B (RhB), a well-established fluorescent probe, was incorporated into the optimized DHCPP gel at a concentration of 0.1% w/w, replacing FTN. This dye served as a surrogate marker due to its inherent ability to emit distinct fluorescence upon excitation (Nemr et al., 2024). At this concentration, no separation step was required, as the dye provided sufficient fluorescence without affecting vesicle integrity (Ahmed et al., 2024a; Ahmed et al., 2025i). Female albino rabbits were randomly assigned into two groups (n = 3 per group). The treatment group received 2 g of RhB-loaded DHCPP gel, whereas the negative control group was administered 2 g of plain RhB gel. Formulations were applied intra-vaginally using a soft polyethylene applicator to ensure uniform coverage and minimize mucosal trauma. After 6 h of exposure, animals were anesthetized with ketamine (35 mg/kg) and xylazine (5 mg/kg), then euthanized following the same previous procedures. The vaginal mucosa was immediately excised, thoroughly rinsed with normal saline and deionized water, and then mounted between a glass slide and coverslip for microscopic analysis (Ahmed et al., 2025a). Fluorescence imaging was conducted using a CLSM (LSM 710, Carl Zeiss, Jena, Germany) equipped with argon and helium–neon lasers at excitation wavelengths of 485 nm and 595 nm, respectively (Ahmed et al., 2022). The resulting confocal images were processed and analyzed using LSM software version 4.2 (Carl Zeiss Microimaging, Jena, Germany) to assess the fluorescence distribution and penetration depth across the vaginal epithelium (Sayed et al., 2021). This study provided direct visual evidence of enhanced mucosal permeation conferred by the DHCPP system, validating the findings from the *ex vivo* diffusion studies and confirming the formulation’s potential for improved intravaginal drug delivery performance.

#### 2.9.4 Histopathological assessment

To assess the local safety and mucosal tolerance of DHCPP gel following vaginal application, a histopathological study was performed using female albino rabbits. The animals were randomly divided into two groups (n = 3 each): a negative control group that received no treatment and a test group treated with the optimized DHCPP gel. The DHCPP gel (equivalent to 1000 µg FTN) was administered intra-vaginally twice daily for 1 week using a soft polyethylene applicator. After treatment termination, the animals were anesthetized and subsequently euthanized. Vaginal tissue specimens were excised and immediately fixed in 10% neutral buffered formalin for 24 h to preserve structural integrity. The samples were then sequentially dehydrated using graded alcohol concentrations (methanol, ethanol, and absolute ethanol), cleared with xylene, and embedded in paraffin wax at 56 °C to obtain tissue blocks. Thin sections (approximately 4 µm) were cut using a rotary microtome, mounted on glass slides, deparaffinized, and stained with hematoxylin and eosin (H&E) following standard histological protocols (Nemr et al., 2025; Abdelbary and AbouGhaly, 2015). The stained sections were examined under a light microscope (CX21, Olympus, Tokyo, Japan) to detect any signs of epithelial damage, edema, inflammatory infiltration, or other histological abnormalities. Comparison with control tissues confirmed the biocompatibility and non-irritant nature of the developed formulation toward the vaginal mucosa (Helal et al., 2025).

### 2.10 Statistical analysis

Statistical evaluation of the experimental design was conducted using one-way analysis of variance (ANOVA) implemented in Design Expert® software (Version 13, Stat-Ease Inc., Minneapolis, MN, USA). All experimental measurements were performed in triplicate (n = 3), and the results are expressed as mean ± standard deviation (SD). When significant differences among groups were observed, post-hoc comparisons were carried out using the Least Significant Difference (LSD) test to determine pairwise variations. A p-value of less than 0.05 was considered statistically significant for all analyses.

## 3 Results and discussion

### 3.1 Factorial model interpretation and response analysis

To achieve a precise optimization of DHCPPs, a 2^3^ factorial design was systematically employed. This design enabled an in-depth investigation of the critical formulation factors influencing the nanocarrier’s physicochemical properties and performance. The selected independent variables were: the DDAB: drug ratio (Factor A), the PEG: DDAB ratio (Factor B), and the PC: PEG ratio (Factor C). Using the factorial matrix, eight experimental runs were generated to evaluate the effects of the studied variables while minimizing experimental workload (Zhang et al., 2010). The responses selected as critical quality attributes included EE%, PDI, ZP and PS. These parameters collectively reflect encapsulation capability, nanoscale uniformity, and electrostatic stability, which are essential for ensuring efficient vaginal delivery. The experimental data were analyzed using Design-Expert® software. Regression modeling and response surface methodology (RSM) were applied to derive polynomial equations, predict optimal conditions, and visualize the influence of each factor on the measured responses (Habib et al., 2018). Statistical validation confirmed the reliability and accuracy of the developed models, as indicated by high coefficients of determination (*R* ^2^) and minimal differences (<0.2) between adjusted and predicted *R* ^2^ values. Furthermore, adequate precision values for all responses exceeded the recommended threshold of 4, signifying strong model predictability and a favorable signal-to-noise ratio (Younes et al., 2024; Younes et al., 2025) (Table 3).

**TABLE 3:** Statistical assessment of the examined responses.

Response	*R* ^2^	Adjusted *R* ^2^	Predicated *R* ^2^	Adequate precision	Significant factors
EE %	0.9585	0.9274	0.8340	13.44	A,C
PS (nm)	0.8813	0.7922	0.7251	9.00	A,B
ZP (mV)	0.9565	0.9239	0.8261	13.93	A, C

Abbreviations: EE%, entrapment efficiency percentage; PS, particle size; ZP, zeta potential.

#### 3.1.1 Statistical evaluation of EE%

**FIGURE 1:**
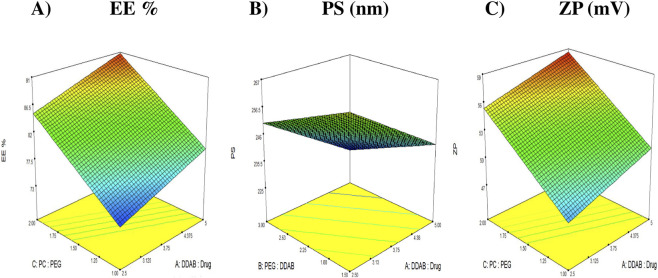
3D response plots illustrating the effect of DDAB: drug ratio (factor X_1_), PEG: DDAB ratio (factor X_2_) and PC: PEG ratio (factor X_3_) on entrapment efficiency (%EE), particle size (PS), and zeta potential (ZP). **(A)** %EE. **(B)** PS (nm). **(C)** ZP (mV).

Efficient drug encapsulation is a critical determinant of nanocarrier performance, directly influencing therapeutic retention, stability, and localized bioavailability (Vanic et al., 2021). The EE% of the prepared Dual-Functional Hybrid Cationic PEGylated Proniosomes (DHCPP) exhibited high values ranging from 72.34% ± 2.37% to 91.61% ± 1.98%, reflecting the system’s strong ability to retain the lipophilic antifungal agent within the vesicular bilayer matrix. The relationship between the formulation variables and EE% was mathematically described by the regression equation:

EE%=82.27+2.82A−0.40B+5.72C

where A, B, and C correspond to the DDAB: drug ratio, PEG: DDAB ratio, and PC: PEG ratio, respectively. Statistical analysis confirmed that factors A and C exerted significant positive effects (*p* < 0.05) on the response, as illustrated in Figure 1A, signifying their pivotal role in optimizing the encapsulation of FTN. While factor B was non-significant (*p >* 0.05).

The DDAB: drug ratio (Factor A) markedly influenced EE%. Increasing the DDAB proportion improved drug retention owing to its pronounced lipophilicity (log P ≈ 11.8) (Carbone et al., 2012), which reinforced the hydrophobic core of the proniosomal bilayer. The long alkyl chains of DDAB enhance bilayer compactness and reduce drug diffusion into the aqueous medium, resulting in superior vesicular integrity. Moreover, the quaternary ammonium head group of DDAB imparted a strong cationic charge that not only stabilized the vesicular surface electrostatically but also improved the organization of the amphiphilic molecules within the lipid phase (Nemr et al., 2025). These combined hydrophobic and electrostatic contributions established a rigid yet cohesive bilayer structure, favoring efficient encapsulation of the lipophilic FTN molecules (Chou et al., 2014; Cooper and Harirforoosh, 2014).

Similarly, the PC: PEG ratio (Factor C) exhibited a positive and statistically significant impact on EE%. Formulations containing higher proportions of phosphatidylcholine (PC) exhibited greater %EE, possibly due to the naturally high phase transition temperature (Tc) of lecithin (Ahmed et al., 2025f). This characteristic reduces bilayer fluidity and permeability, thereby minimizing drug leakage and effectively preserving the encapsulated cargo (Emad Eldeeb et al., 2019; Sayed et al., 2020). In addition, the amphiphilic nature of PC, with hydrophobic tails forming the core and hydrophilic phosphate head groups aligning toward the aqueous interface, provides a stable, cohesive bilayer that accommodates lipophilic drugs efficiently (Abdelbary et al., 2017; Abdelbary and Aburahma, 2015). The strong hydrophobic interactions between PC and DDAB tails reinforce bilayer compactness, while hydrogen bonding between PC and PEG chains contributes to interfacial flexibility and steric stabilization. In summary, both DDAB and PC play synergistic roles in establishing a stable and hydrophobic bilayer architecture, while PEG enhances colloidal stability and prolongs vesicular lifespan.

#### 3.1.2 Statistical evaluation of PS

Particle size (PS) plays a decisive role in determining the biological behavior, diffusion dynamics, and therapeutic efficiency of nanosystems intended for vaginal delivery. Maintaining an optimal nanoscale dimension ensures intimate contact with the mucosal surface, deeper tissue penetration, and improved drug retention within the target site (Ahmed et al., 2025a). In the present study, the particle size of the prepared DHCPPs ranged from 226.7 ± 3.04 to 256.1 ± 2.33 nm, confirming the successful fabrication of homogeneously dispersed vesicles with suitable dimensions for mucosal application. Statistical analysis revealed that factors A and B exerted significant negative effects (*p* < 0.05), indicating their prominent roles in governing vesicular size (Figure 1B). While factor C was non-significant (*p >* 0.05). The relationship between the independent variables and the observed PS values was expressed by the following regression equation:

PS=246.47−12.33A−8.20B−5.64C

The DDAB: drug ratio (Factor A) demonstrated a pronounced inverse relationship with PS, where increasing the DDAB proportion led to a reduction in vesicle diameter. This downsizing can be attributed to the strong stabilizing influence of DDAB, which enhances bilayer cohesion through its cationic quaternary ammonium head group and long hydrophobic alkyl chain. The resulting electrostatic repulsion between positively charged vesicles minimizes aggregation, promoting the formation of discrete, well-separated nanoparticles (Carbone et al., 2014). Moreover, DDAB’s inherent lipophilicity (log P ≈ 11.8) contributes to a more compact lipid arrangement, decreasing inter-vesicular fusion and yielding smaller, uniformly distributed particles. Similar findings were reported by Cooper et al., who observed that DDAB incorporation into celecoxib-loaded PLGA nanoparticles produced smaller, more stable systems with higher encapsulation efficiency (Cooper and Harirforoosh, 2014). Beyond size reduction, this phenomenon also supports improved drug loading, as smaller vesicles often possess greater surface area and tighter packing density, favoring retention of hydrophobic drugs within the bilayer.

The PEG: DDAB ratio (Factor B) also exhibited a significant negative effect on particle size, further reinforcing the crucial stabilizing role of PEGylation in vesicle formation. PEG reduces interfacial tension between the aqueous and lipid phases, facilitating vesicle curvature and spontaneous nanoscale assembly (Fahmy et al., 2025). Increasing PEG content leads to the development of a steric stabilization layer around the vesicles, effectively preventing coalescence and aggregation. This surface coating provides hydration repulsion forces that preserve vesicular individuality and sustain smaller particle dimensions (Zeb et al., 2016). At higher PEG ratios, this effect becomes more prominent, whereas at lower concentrations, incomplete surface coverage may lead to partial aggregation and consequently larger vesicles (Yousry et al., 2016). Comparable outcomes were reported by and Kim et al. (2009), and Wolfram et al. (2014), who observed that PEGylation markedly decreased the vesicular size of the formulated systems. Collectively, the synergistic contribution of DDAB and PEG results in a balance between electrostatic and steric stabilization forces that finely control vesicle size.

#### 3.1.3 Statistical evaluation of PDI

The poly-dispersity index (PDI) serves as a critical indicator of nanoparticle uniformity, reflecting the degree of homogeneity in size distribution and, consequently, the reproducibility and stability of the formulation. In general, PDI values approaching zero signify a highly monodisperse system with uniform vesicle populations, whereas values nearing one indicate heterogeneous dispersions with wide size variability (Danaei et al., 2018). For pharmaceutical nanocarriers, PDI values below 0.5 are typically deemed acceptable, denoting well-controlled size distribution that supports consistent drug release, and stable behavior during storage and administration (Nemr et al., 2021).

In the present study, the prepared formulations demonstrated PDI values ranging between 0.20 ± 0.01 and 0.27 ± 0.03, confirming their narrow particle size distribution. Statistical analysis revealed that none of the investigated formulation factors (A, B, or C) exerted a statistically significant effect on PDI values (*p >* 0.05). Although the PDI model was thus excluded from the optimization criteria due to its lack of sensitivity to variable changes, the consistently favorable results underscore the robustness and reproducibility of the fabrication process.

#### 3.1.4 Statistical evaluation of ZP

Zeta potential (ZP) is a fundamental indicator of the electrostatic stability of nanocarrier systems, as it reflects the degree of repulsion between dispersed particles and, consequently, their tendency to aggregate. Nanovesicles with surface charges exceeding ±20 mV are typically considered electrostatically stable, since strong repulsive forces prevent particle flocculation and ensure a well-dispersed colloidal system (El et al., 2023). In the present study, the formulated DHCPPs exhibited ZP values ranging from 45.50 ± 0.57 mV to 58.85 ± 2.47 mV (Table 2), confirming excellent electrostatic stability throughout the design space. As illustrated in Figure 1C, factor A (DDAB: drug ratio) and factor C (PC: PEG ratio), showed statistically significant positive effects (*p <* 0.05) on the zeta potential, indicating that increasing either factor enhanced the surface charge of the nanovesicles. While factor B was non-significant (*p >* 0.05). The statistical model describing the relationship between formulation factors and ZP was expressed by the following coded equation:

ZP=53.27+1.61A – 0.83B+3.77C

The positive impact of Factor A can be attributed to the intrinsic cationic nature of DDAB. The quaternary ammonium head groups of DDAB contribute to the generation of a stronger positive surface potential as their proportion increases. This effect arises from the higher density of positively charged sites on the vesicular surface, which enhances electrostatic repulsion between particles, minimizing aggregation and improving overall dispersion stability (Albash et al., 2021; Nemr et al., 2025). Moreover, the presence of DDAB promotes ordered molecular packing within the bilayer structure, strengthening interfacial organization and contributing to enhanced vesicle rigidity and physical stability (Gossmann et al., 2015).

The significant positive effect of Factor C (PC: PEG ratio) can be explained by the amphiphilic and structural characteristics of PC. As PC concentration increases relative to PEG, the lipid bilayer becomes more compact and densely packed, leading to enhanced exposure of positively oriented head groups on the vesicle surface (Younes et al., 2024). This contributes to an increase in the overall surface potential, further stabilizing the formulation. A higher PC content also strengthens the bilayer’s mechanical integrity, reducing the risk of vesicle fusion and drug leakage (Ahmed et al., 2025f). Although PEGylation may provide steric stabilization by creating a hydration shell around vesicles, it does not directly affect electrostatic interactions in this system.

### 3.2 Selection of optimum DHCPP

Optimization of the developed DHCPPs was carried out using Design-Expert® software (version 13, Stat-Ease Inc., Minneapolis, USA). The software employed a multi-response desirability approach, integrating all critical formulation responses into a single composite index to identify the most favorable formulation conditions (Helal et al., 2024). Among the evaluated responses, encapsulation efficiency (EE%), particle size (PS), and zeta potential (ZP) were chosen as the key quality attributes, as they collectively determine the vesicular integrity, stability, and drug delivery efficiency. The poly-dispersity index (PDI) was excluded from the optimization model due to its consistently low values, confirming homogeneity of vesicle distribution across all experimental runs. Based on the software-generated model, the optimal formulation was identified at the factor levels of DDAB: drug ratio (A) = 5:1, PEG: DDAB ratio (B) = 1.5:1, and PC: PEG ratio (C) = 2:1, achieving an overall desirability score of 0.931.

The optimized formulation exhibited experimental values in close agreement with the predicted ones, confirming the validity and reliability of the model (Table 4) (Ahmed et al., 2025e). The final formulation displayed an encapsulation efficiency of 91.61% ± 1.98%, a mean particle size of 238.85 ± 3.46 nm, a PDI of 0.22 ± 0.01 and a zeta potential of +58.85 ± 2.47 mV, indicating the formation of stable, nano-sized vesicles with strong electrostatic repulsion and efficient drug retention. The high positive surface potential further suggested excellent colloidal stability, minimizing aggregation and ensuring prolonged mucosal residence. Therefore, the aforementioned formulation was further evaluated to assess its efficacy for the underlying treatment condition.

**TABLE 4:** The predicted and actual outcomes of the optimized Hybrid Cationic PEGylated Proniosomes.

Response	Y1	Y2	Y4
	EE %	PS (nm)	ZP (mV)
Observed value	91.61	238.85	58.85
Predicated value	91.20	236.54	59.46
% bias (absolute)	0.45	0.97	1.02

Abbreviations: EE %, entrapment efficiency percentage; PS, particle size; ZP, zeta potential.

### 3.3 Characterization of the optimum DHCPP

#### 3.3.1 TEM visualization

**FIGURE 2:**
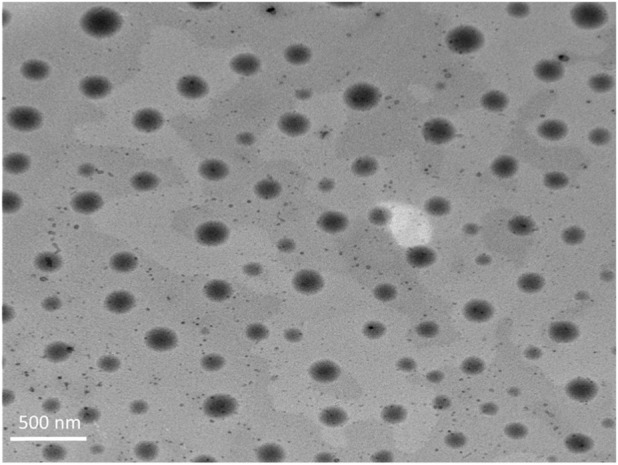
TEM images of the optimum formula showing the spherical vesicles, lacking any sort of aggregation.

TEM was employed to visualize the morphological characteristics of the optimized DHCPP. The obtained micrograph (Figure 2) revealed well-defined, spherical vesicles with smooth surfaces and uniform size distribution, confirming the successful formation of nanoscale DHCPP. The vesicles appeared distinctly separated and non-agglomerated, reflecting effective stabilization by the incorporated PEG, DDAB and phosphatidylcholine components, which provided both steric and electrostatic repulsion (Fahmy et al., 2025; Tawfik et al., 2025).

#### 3.3.2 FT-IR analysis

The effect of lyophilization on the optimized formulation was evaluated by comparing particle size and poly-dispersity index (PDI) before and after freeze-drying. Statistical analysis demonstrated no significant difference in particle size or PDI (p > 0.05), indicating that the lyophilization process did not adversely affect the nanoscale characteristics or size uniformity of the formulation.

**FIGURE 3:**
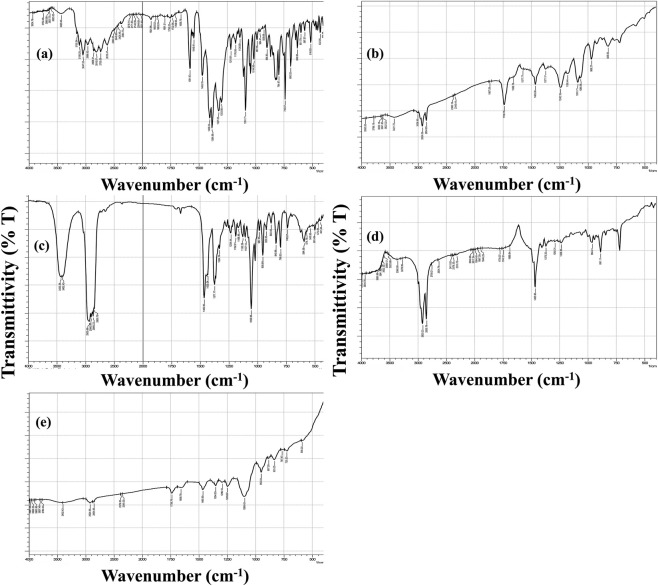
FT-IR spectra of **(a)** fenticonazole, **(b)** phosphatidylcholine, **(c)** cholesterol, **(d)** didodecyldimethylammonium bromide (DDAB), and **(e)** the optimum DHCPP.

The FTIR spectra of the individual components and the optimized DHCPP are presented in Figure 3.

The spectrum of pure FTN (Figure 3a) exhibited distinct and sharp absorption bands characteristic of its molecular structure. The prominent peak observed at 3047 cm^-1^ corresponds to N–H stretching vibrations, while the band at 1581 cm^-1^ is attributed to the stretching of the imine (C=N) group. Additional peaks appearing at 1469 cm^-1^ and 1091 cm^-1^ were assigned to C=C stretching and aromatic ring vibrations, respectively, consistent with previously reported findings (Ahmed et al., 2022; Castro et al., 2016).

The FTIR spectrum of phosphatidylcholine (PC) (Figure 3b) closely matched the literature profile (Ahmed et al., 2025f). A broad absorption band at 3421 cm^-1^ represented the O–H stretching of the carboxylic group, whereas a strong signal at 1739 cm^-1^ confirmed the presence of ester carbonyl (C=O) stretching. Furthermore, the peaks at 2,924 cm^-1^ and 2,854 cm^-1^ corresponded to the asymmetric and symmetric stretching vibrations of aliphatic C–H bonds, respectively, while the band at 1465 cm^-1^ was linked to aromatic ring vibrations (Younes et al., 2024). Similarly, the cholesterol (CH) spectrum (Figure 3c) displayed a broad peak near 3433 cm^-1^, indicative of the phenolic O–H stretching, accompanied by two overlapping bands at 2,935 cm^-1^ and 2,900 cm^-1^ corresponding to C–H stretching vibrations (Romano et al., 2024). The FTIR spectrum of DDAB (Figure 3d) displayed characteristic vibrational bands corresponding to its aliphatic structure. Prominent methylene-associated peaks appeared at 721, 886, and 1467 cm^-1^, while the symmetric and asymmetric stretching vibrations of C–H bonds were clearly observed at approximately 2,852 and 2,919 cm^-1^, respectively (Mishra et al., 2017). In contrast, the FTIR spectrum of the optimized DHCPP (Figure 3e) demonstrated significant attenuation of FTN’s distinctive peaks. The marked reduction in the intensity of characteristic FTN absorption bands suggests that the drug was successfully encapsulated within the proniosomal bilayer rather than existing in its crystalline form (Sharma et al., 2023).

#### 3.3.3 *In-vitro* release study

**FIGURE 4:**
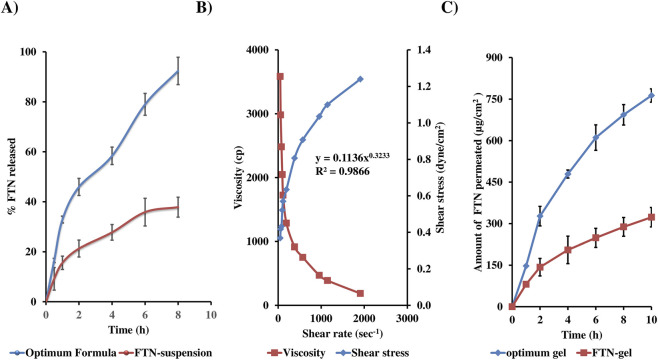
*In vitro* release **(A)**, rheological characteristics **(B)** and *ex-vivo* permeation **(C)** profiles from optimum formula, demonstrating biphasic drug release, pseudo-plastic (shear-thinning) rheology, and enhanced vaginal permeation.

The *in-vitro* release profiles of the optimized DHCPP and the FTN suspension are illustrated in Figure 4A. The optimized formulation exhibited a distinctive biphasic release pattern, typical of proniosomal systems (Alshora et al., 2023; Darson et al., 2023). An initial burst phase was observed within the first 2 hours, attributed to the rapid diffusion of surface-associated FTN molecules, followed by a sustained release phase extending throughout the remainder of the study. This sustained profile reflects the gradual diffusion of the drug from the inner lipidic core of the vesicles, demonstrating the ability of the proniosomal matrix to control and prolong drug release (Younes et al., 2024).

Quantitatively, the optimized DHCPP achieved a markedly higher release compared to the FTN suspension, with Q_2_h = 45.93 ± 3.41% versus 21.27% ± 3.37%, and Q_8_h = 92.36 ± 5.46% versus 37.82% ± 3.99%, respectively. This pronounced enhancement can be attributed to the improved solubilization capacity and nanoscale encapsulation within the proniosomal system, which increases the apparent solubility and diffusion rate of the otherwise poorly water-soluble FTN (solubility <0.1 mg/mL) (Khudair et al., 2020). Kinetic modeling of the release data revealed that the profile best fitted the Higuchi diffusion model (*R* ^2^ = 0.987), confirming that FTN release from DHCPP was predominantly diffusion-controlled. These findings substantiate the formulation’s capacity to provide an initial therapeutic burst followed by a sustained, controlled release that is required for enhancing and maintaining prolonged antifungal efficacy at the vaginal site.

#### 3.3.4 Mucoadhesion study

The mucoadhesive potential of the optimized DHCPP was thoroughly investigated to evaluate its ability to interact with and remain attached to the vaginal mucosal surface to ensure localized, prolonged antifungal action. Since vaginal mucosa is rich in negatively charged mucin glycoproteins, its interaction with the cationic DHCPP vesicles was examined using mucin dispersion as a biological mimic of the mucosal environment (Ahmed et al., 2025d). Upon mixing the DHCPP formulation with mucin, a marked reduction in zeta potential was observed, signifying the formation of electrostatic complexes between the positively charged vesicles and the negatively charged mucin chains. This interaction confirms the establishment of strong electrostatic bonding forces, leading to the adsorption of mucin onto the vesicular surface (Tawfik et al., 2025). The high positive charge imparted by DDAB, combined with the stabilizing PEG coating, not only strengthened these electrostatic attractions but also created a hydrated interfacial layer that enhances mechanical adhesion (Ahmed et al., 2025b).

#### 3.3.5 Short term stability study

The short-term stability of the optimized DHCPP was systematically assessed to ensure the preservation of its physicochemical and functional integrity during storage. The formulation was stored under refrigerated conditions (4 °C ± 2 °C) for a period of 3 months. Post-storage analysis revealed EE% of 89.21% ± 0.67%, PS of 237.75 ± 3.32 nm, and ZP of 51.35 ± 4.45 mV, which were statistically comparable (*p >* 0.05) to those of the freshly prepared sample (EE% = 91.83 ± 1.11%, PS = 234.55 ± 3.61 nm, ZP = 56.9 ± 2.4 mV). The negligible variations indicate excellent formulation stability, with minimal drug leakage, aggregation, or vesicle coalescence during storage (Hegazy et al., 2025). Such stability can be primarily attributed to the protective effect of PEGylation, which provides a steric barrier on the vesicular surface, reducing particle–particle interaction and preventing aggregation (El-Naggar et al., 2026). Additionally, the cationic nature of DDAB reinforces electrostatic repulsion, contributing to the formulation’s resistance against structural destabilization and fusion over time (Nemr et al., 2025).

Furthermore, a comparative evaluation of the *in-vitro* release profiles between the fresh and stored samples yielded an f_2_ similarity factor of 73.73, confirming the *in-vitro* equivalence. The absence of significant differences in Q_8_h values (87.40% ± 4.32% vs. 92.36% ± 5.46%) further corroborates that storage did not adversely affect the drug release kinetics or diffusion behavior (Diaz et al., 2016).

### 3.4 Microbiological assay

#### 3.4.1 Determination of MIC

The MIC assay was performed using the broth microdilution technique to quantitatively assess the antifungal potency of the optimized DHCPP and its plain suspension against *C. albicans*. This evaluation aimed to confirm whether the DHCPP enhanced the antifungal efficacy of FTN compared to its plain suspension. Remarkably, the optimized DHCPP demonstrated a MIC value of 0.244 μg/mL, which was approximately 64-fold lower than that of the FTN suspension (15.6 μg/mL). This pronounced improvement underscores the significant potentiation of antifungal activity achieved through nanoscale encapsulation. The enhanced performance can be attributed to multiple synergistic mechanisms: first, the nano-sized vesicular structure increases the surface area available for fungal cell interaction; second, the positive surface charge promotes electrostatic attraction with the negatively charged fungal cell wall, thereby facilitating enhanced adhesion and membrane permeation; and third, the PEGylated bilayer composition ensures greater stability and prolonged drug availability at the infection site, enabling sustained antifungal action.

Furthermore, the improved drug solubilization and dispersion within the vesicular matrix likely increased FTN’s effective concentration at the fungal cell interface, leading to better inhibition of ergosterol biosynthesis and disruption of membrane integrity. These findings collectively confirm that the optimized DHCPP not only enhances drug delivery efficiency but also amplifies antifungal efficacy, positioning it as a promising therapeutic strategy for the management of vaginal candidiasis, especially in resistant or recurrent cases.

#### 3.4.2 Determination of MFC

Similarly, the MFC values further reinforced these findings. Both the optimized DHCPP and the FTN suspension exhibited fungicidal activity after 48 h of incubation at 28 °C ± 2 °C; however, the optimized DHCPP displayed a significantly lower MFC (1.953 μg/mL) compared to the drug suspension (62.5 μg/mL). This notable difference reflects the improved fungicidal capability of the nanosystem, which can be linked to its enhanced interaction with fungal membranes and more efficient drug delivery to intracellular targets responsible for cell death.

#### 3.4.3 Determination of biofilm inhibitory effect

**FIGURE 5:**
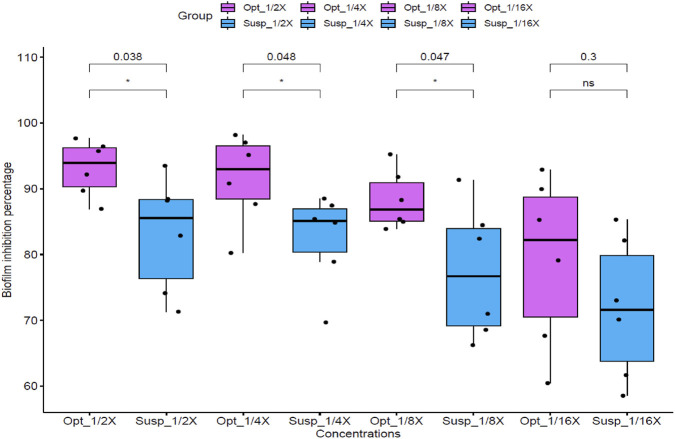
Anti-biofilm activity of optimized formula (Opt) and FTN suspension (Susp) against *Candida albicans* standard strain ATCC 60193 at different concentrations (^1^/_16,_^1^/_8_, ¼, and ½ X, where X is the calculated MIC). Optimized formula concentrations are as follows: ^1^/_16_ X = 0.015 μg/mL, ^1^/_8_ X = 0.0305 μg/mL, ^1^/_4_ X = 0.061 μg/mL, ^1^/_2_ X = 0.122 μg/mL. FTN suspension concentrations are as follows: ^1^/_16_ X = 0.975 μg/mL, ^1^/_8_ X = 1.95 μg/mL, ^1^/_4_ X = 3.9 μg/mL, ^1^/_2_ X = 7.8 μg/mL. Statistical significance was assessed, within each concentration between optimized formula and FTN suspension, by Student’s t-test, and all *p* values are shown.

The crystal violet staining method was employed to assess and compare the biofilm inhibition potential of the optimized DHCPP and the FTN suspension. This assay was conducted using *C. albicans* as a representative fungal strain, focusing on sub-inhibitory concentrations corresponding to ^1^/_16_, ^1^/_8_, ¼, and ½ X, where X denotes the previously determined MIC value. As illustrated in Figure 5, the optimized DHCPP exhibited a remarkably stronger anti-biofilm effect compared to the free drug suspension, particularly at ^1^/_8_, ¼, and ½ MIC concentrations (*p <* 0.05). These results clearly demonstrate the enhanced ability of the optimized DHCPP to prevent biofilm formation at concentrations below the minimal inhibitory threshold. The pronounced improvement in biofilm inhibition may be attributed to the superior mucoadhesive characteristics and nanoscale architecture of the proniosomal system, which facilitate intimate interaction with the fungal surface and prolonged drug retention within the biofilm microenvironment. Moreover, the cationic nature of DDAB promotes electrostatic binding to the negatively charged fungal cell membrane, leading to disruption of the biofilm matrix and hindering fungal adhesion and colonization. At the lowest tested concentration (^1^/_16_ MIC), no statistically significant difference (*p >* 0.05) was observed, indicating that at extremely low drug levels, the available concentration of active molecules might be insufficient to achieve effective biofilm disruption.

### 3.5 Development and characterization of optimum DHCPP gel

#### 3.5.1 Rheological study

Looking at the rheogram in Figure 4B, it is evident that as the shear rate increased, a fall in viscosity was noticed, suggestive of a shear-thinning behavior. This shear-thinning characteristic is indicative of non-newtonian flow behavior, confirmed by the power law index (n) of 0.3233 (Ansari et al., 2020). Such flow property is not only suitable for easy application of the gel within the vaginal cavity, but also with maintaining the gel *in-situ* to ensure prolonged drug release. Moreover, based on the *R* ^2^ of the investigated models, the optimum DHCPP gel was found to follow Carreau’s model.

#### 3.5.2 pH measurement

The measured pH of the prepared gel was 4.21 ± 0.24, placing it comfortably within the physiological vaginal range of 4.0–4.5. Maintaining this acidic environment is crucial, as it aligns with the natural vaginal pH, thereby supporting mucosal compatibility while minimizing the risk of irritation, inflammation, or microbial imbalance. Such a pH ensures the formulation’s suitability for safe and well-tolerated vaginal application (El-Naggar et al., 2026).

### 3.6 Animal studies

#### 3.6.1 *Ex-vivo* permeation study

During the *ex vivo* permeation study, a lag time of approximately 1 h was observed before the onset of drug permeation. Several findings can be interpreted from Figure 4C, illustrating the cumulative permeation profiles of the DHCPP gel and FTN suspension. Evidently, an increase in the amount of FTN permeated can be seen from the DHCPP gel as compared to the control FTN gel (763.1 ± 23.97 vs. 323.21 ± 34.99 $\mu g/{\text{cm}}^{2}$ , respectively). Furthermore, parameters such as the drug flux ( $J_{\max}$ ) and ER showcased similar enhancements. The DHCPP gel possessed a flux ( $J_{\max}$ ) of 76.31 ± 2.4 $\mu g/{\text{cm}}^{2}/h$ which is significantly (*p* < 0.05) larger than that of the FTN gel (32.32 ± 3.5 $\mu g/{\text{cm}}^{2}/h$ ). Ultimately, the DHCPP gel exhibited an ER of 2.36 compared to the FTN gel. DHCPP undergo hydration in the aqueous environment *in-situ* to form niosomes that allow for increased surface area, biological membrane contact, and drug permeation and uptake through the membrane (Zimmermann et al., 2021). PC, exhibiting structure similarity with biological membranes, acts as penetration enhancer (Harasym and Banas, 2024). PEG adds up to the permeability enhancement owing to its positive effects on membrane porosity and flexibility (Salahuddin et al., 2018), Furthermore, the formulation’s mucoadhesive properties and sustained release behaviour support drug retention within the target tissue.

#### 3.6.2 Confocal laser scanning microscopy (CLSM)

**FIGURE 6:**
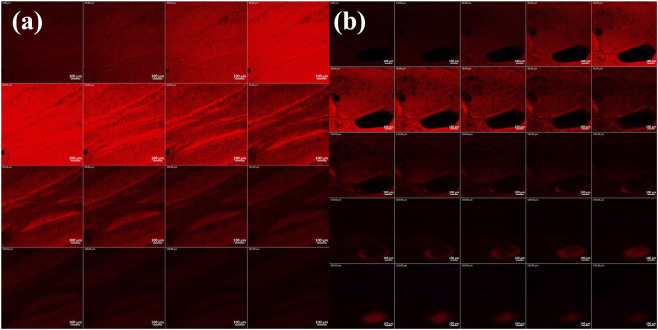
Confocal laser scanning microscopic images of **(a)** rhodamine B aqueous solution and **(b)** Rhodamine B-loaded DHCPP gel. The images suggest the deeper penetration of the DHCPP gel.

To further consolidate the findings of *ex-vivo* permeation, CLSM was carried. The microscopy images in Figure 6 indicate the higher penetrability of RhB from the prepared DHCPP gel (240 μm ), in comparison to that from its plain RhB gel (control group, 150 μm ). As seen in *ex-vivo* permeation, the enhanced penetrability through vaginal mucosa can be attributed to the successful hydration of proniosomes to form niosomes *in-situ*. Niosomes, being nanoparticles, are characterized by small PS and large surface area which increase contact area and cellular uptake. Additionally, PC and PEG exert synergistic effects to the positive permeation effects perceived.

#### 3.6.3 Histopathological assessment

**FIGURE 7:**
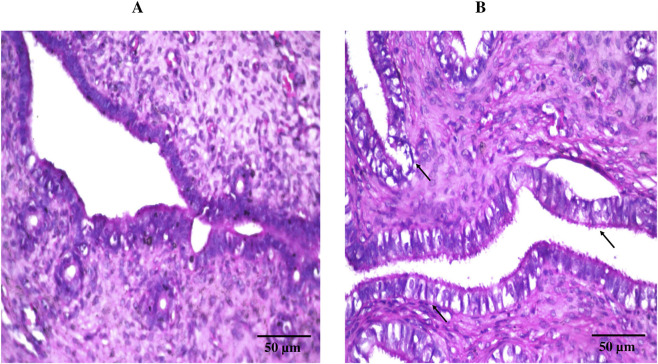
Histopathological images of **(A)** control group (left untreated) and **(B)** treatment group (DHCPP gel), showing lack of alteration in normal tissue structure in both groups following staining with hematoxylin and eosin.

Consistent with the previously observed pH findings, histological evaluation using hematoxylin and eosin staining (Figure 7) revealed comparable results between the treatment (DHCPP gel) and control groups. The control group (Figure 7A) displayed normal vaginal mucosal architecture, characterized by healthy epithelial cells, well-differentiated epithelium, and intact connective tissue beneath. Similarly, the treatment group (Figure 7B) exhibited a histological appearance closely resembling that of the control, with no noticeable tissue alterations. These findings, in harmony with the pH observed, further support the biocompatibility and safety of the applied DHCPP gel.

## 4 Limitation of the study

Although the current animal study provided valuable insights, inclusion of a larger number of animals could have further increased the statistical power of the findings. However, the study intentionally employed a minimal number of animals to adhere to the 3R principles of animal research (reduce, reuse, and recycle), ensuring ethical compliance while maintaining reliable and reproducible results.

## 5 Conclusion

The present investigation successfully introduced and optimized Dual-Functional Hybrid Cationic PEGylated Proniosomes (DHCPP) as an innovative nano-platform for the localized management of vaginal candidiasis. The strategic integration of PEGylation and cationic components within a hybrid proniosomal matrix conferred synergistic advantages, resulting in enhanced physicochemical stability, efficient mucosal interaction, and improved biological performance. The optimized formulation, selected using a factorial design with a high desirability value (0.931), exhibited superior encapsulation efficiency, nanoscale particle size, and a highly positive zeta potential, collectively ensuring colloidal stability and effective interaction with the negatively charged vaginal mucosa.

Comprehensive *in vitro*, *ex vivo*, microbiological, and *in vivo* evaluations confirmed the multifunctional nature of the DHCPP system. Enhanced mucoadhesion and pseudo-plastic gel behavior promoted prolonged vaginal residence and improved local drug retention, while diffusion-controlled drug release enabled sustained antifungal activity. FTIR analysis verified molecular compatibility and successful encapsulation of FTN. Notably, the significant enhancement in vaginal permeation, pronounced biofilm inhibition, and marked reductions in MIC and MFC values highlight the system’s ability to enhance antifungal efficacy and potentially reduce infection recurrence. Histopathological examination further confirmed the safety, tolerability, and biocompatibility of the optimized gel following repeated administration.

Importantly, the clinical relevance of the developed DHCPP system is underscored when compared with conventional topical FTN formulations. Beyond improved vaginal permeation, the DHCPP system offers clinically meaningful advantages, including enhanced mucoadhesion resulting in prolonged vaginal residence time, controlled and sustained drug release, and superior antifungal performance. These attributes may translate clinically into reduced dosing frequency, improved therapeutic outcomes, and enhanced patient compliance. Moreover, incorporation of the optimized DHCPP into a pH-compatible, non-irritant carbopol gel supports local safety and comfort, as confirmed by histopathological findings.

Collectively, these features highlight the feasibility, clinical applicability, and therapeutic utility of the developed DHCPP system, positioning it as a promising alternative to conventional FTN topical products for the effective management of vaginal candidiasis.

## Data Availability

The raw data supporting the conclusions of this article will be made available by the authors, without undue reservation.
