# Supplementary material for: Innovative dual-functional hybrid cationic PEGylated proniosomes as a smart nano-platform for Boosted vaginal delivery: multi-level in-vitro, ex-vivo, microbiological, and in-vivo studies
Source: Front Pharmacol. 2026 Jan 28;16:1746918. doi: 10.3389/fphar.2025.1746918 (PMC12891214; doi:10.3389/fphar.2025.1746918)
Supplement: Supplementary file 1 [file Table1.docx]

**Supplementary Material:** General analytical approaches reported for the quantification of Fenticonazole nitrate in aqueous and non-aqueous samples

| **Sample type** | **Solubility of FTN** | **Solubilization approach** | **Analytical technique** | **Typical analytical conditions** |
| --- | --- | --- | --- | --- |
| **Aqueous samples** | Practically insoluble in water (< 0.1 mg/mL) | Addition of organic solvent and/or surfactant system to achieve solubility | UV–spectrophotometry | Detection at λmax ≈ 252 nm after suitable dilution |
|  |  | Use of aqueous/organic mobile phase | HPLC | Methanol–water (85:15, v/v); flow rate 1.2 mL/min; injection volume 20 μL; detection at 252 nm |
| **Non-aqueous samples** | Freely soluble in organic solvents | Direct dissolution in organic solvent (e.g., methanol) | UV–spectrophotometry | Detection at λmax ≈ 252 nm |
|  |  | Organic solvent as mobile phase | HPLC | Methanol as organic mobile phase; UV detection at 252 nm |
